# From PCV1 to “PCV5”: Genetic Diversity, Evolutionary Dynamics, and Host Adaptation of Porcine Circoviruses

**DOI:** 10.3390/microorganisms14102313

**Published:** 2026-10-10

**Authors:** Wanting Yu, Chuqi Liao, Xinnuo Lei, Huaping Zheng, Yixuan Shu, Zhihao Wang, Ruping Nie, Yishan Liu, Jiabei Luo, Chaoying Sun

**Affiliations:** 1 College of Animal Science and Technology, Jiangxi Agricultural University, Nanchang 330045, China; 19870754070@163.com (C.L.); 13979194617@163.com (H.Z.); syxmail2006@163.com (Y.S.); 15932922305@163.com (Z.W.); nieruping2025@163.com (R.N.); cc007524@163.com (Y.L.); 19870665610@163.com (J.L.); 2 Jiangsu Key Laboratory for High-Tech Research and Development of Veterinary Biopharmaceuticals, Engineering Technology Research Center for Modern Animal Science and Novel Veterinary Pharmaceutic Development, Jiangsu Agri-Animal Husbandry Vocational College, Taizhou 225300, China; xlei@jsahvc.edu.cn; 3 Animal Production and Health Laboratory, Joint FAO/IAEA Centre of Nuclear Techniques in Food and Agriculture, International Atomic Energy Agency, 2444 Seibersdorf, Austria; suncypku@gmail.com

**Keywords:** porcine circovirus, PCV5, CRESS DNA virus, comparative genomics, cross-species transmission, host adaptation

## Abstract

Porcine circoviruses (PCVs) are among the smallest animal viruses that replicate autonomously. What began in 1974 as a single cell culture contaminant, PCV1, now counts five members, PCV1 to PCV5. The newest of these, PCV5, was reported in 2025. Its genome is larger than those of the other four (2904 nt), and it bears almost no sequence resemblance to any swine-origin circovirus described so far, with only 26.2% amino acid identity for Rep and 20.8% for Cap. Instead, it clusters with fur seal feces-associated circular DNA virus, a lineage that has been proposed for reassignment to the genus *Macochavirus*. In this review we set the genomes of PCV1 to PCV5 side by side, together with the domain organization of their Rep and Cap proteins and their phylogenetic relationships, and we then turn to evolutionary rates, genetic diversity, host adaptation, and cross-species transmission, read against host immune pressure, intensive swine production, and the global live-pig trade. Our reading of the evidence is that these viruses do not share one ancestor. PCV1 and PCV2 descend from a single lineage long adapted to the genus *Sus scrofa*; PCV3 reflects an independent spillover event; the origin of PCV4 is still unresolved; and PCV5 clusters with a fur seal feces-associated circular Rep-encoding single-stranded (CRESS) DNA virus, a phylogenetic affinity consistent with—but at this stage only a testable hypothesis of—an origin at the marine–terrestrial interface. If that is correct, the PCVs are a series of independent spillovers that happened to converge on the same host species, and we argue on that basis for proactive surveillance at the human–livestock–wildlife–environment interface.

## 1. Introduction

### 1.1. History of Discovery and Taxonomic Evolution

The recognition of porcine circoviruses has tracked the development of molecular virology. In 1974, Tischer et al. observed a non-cytopathic contaminant in the continuous porcine kidney cell line PK-15 [1]. By 1982, this agent was shown to carry a covalently closed, circular single-stranded DNA genome and was among the smallest viruses known at the time; it was designated porcine circovirus type 1 (PCV1) [2]. In 1997, Meehan et al. determined the full genome sequence of PCV1 and reported, for the first time, its phylogenetic relationship with plant circoviruses [3]. Because it is not pathogenic in swine, PCV1 was long regarded as little more than a laboratory curiosity [4]. In the mid-to-late 1990s, outbreaks of postweaning multisystemic wasting syndrome (PMWS) were reported successively in North America and Europe. In 1998, Ellis et al. isolated a novel circovirus from lesions of pigs with PMWS [5], and subsequent work showed that this virus was antigenically distinct from PCV1, establishing porcine circovirus type 2 (PCV2) as the causative agent of PMWS [4,6]. Porcine circovirus-associated disease has since become one of the most costly disease problems facing the global swine industry [7].

Discoveries have since accelerated. In 2016, two independent groups in the United States identified porcine circovirus type 3 in pigs with porcine dermatitis and nephropathy syndrome [8,9], cardiac and multisystemic inflammation, and reproductive failure. Researchers in China confirmed the virus in several provinces shortly afterwards [10,11], and detections across Asia, Europe, and the Americas eventually settled the name PCV3, which is still the one in general use [12,13]. In 2019, Chinese scientists detected PCV4 in clinical samples from Hunan Province [14], and the virus was later reported in South Korea [15,16] and Thailand [17]. In 2025, Liu et al. at South China Agricultural University reported a novel porcine circovirus, PCV5 (a vernacular designation retained pending ICTV ratification of the proposed reclassification; see Section below), detected in piglets with respiratory disease and diarrhea and in sows with reproductive disorders, in *Microbiology Spectrum* [18]. In 2026, a team at Henan Agricultural University independently detected and sequenced PCV5 from diagnostic clinical samples across seven provinces in central and eastern China [19]. Also in 2026, phylogenetic analyses of complete genomes and encoded proteins led to a proposal to reclassify FSfaCV/PCV5-related viruses as a porcine-origin lineage within the genus *Macochavirus* [20]. What began as a single virus species has thus become a highly diverse group that almost certainly does not share a monophyletic origin; the full argument is assembled in Section 2.4 and Section 5.3.

Taxonomically, the family *Circoviridae* belongs to the phylum *Cressdnaviricota* and contains only two genera, *Circovirus* and *Cyclovirus* [21], which accommodated 65 and 90 species, respectively, as of the 2024 taxonomic update [22]. Species in this family are demarcated at a pairwise whole-genome sequence identity of 80% [21,22]. PCV5, however, does not fall phylogenetically within *Circoviridae*; its name reflects only its host species and genomic organization [18], which in itself challenges both the current taxonomic framework and the term porcine circovirus. Wang et al. took the question up in 2026, comparing ten full-length FSfaCV/PCV5-related genomes. Trees inferred from whole genomes and trees inferred from encoded proteins gave the same answer. The clade fell outside *Circoviridae* and instead sat within the genus *Macochavirus*, family *Pecoviridae*, as a swine-associated lineage of its own. Their sequence identity and genetic distance calculations treated it as a coherent group, and they proposed reclassifying the virus known as PCV5 as “porcine *Macochavirus*” [20]. They also noted that the Rep region of these viruses is more divergent than the Cap region [20]. Should the ICTV accept the proposal, the name PCV5 will become a term of history. For now the proposal remains precisely that. The genus *Macochavirus* has been ratified within the family *Pecoviridae* (order *Ringavirales*) but currently comprises only two recognized species, *Macochavirus kuci* and *Macochavirus uywa*; neither FSfaCV nor the PCV5 lineage has been formally assigned to it. PCV5 therefore remains a provisional, host-based designation rather than an ICTV-recognized virus species. The question underneath it, of how a lineage so close to marine mammal-associated viruses became established in swine, will not go away.

### 1.2. Discovery of PCV5

PCV5 has drawn wide attention because it breaks several assumptions about porcine circoviruses that had held for decades. First, its genome is much larger: whereas PCV1–PCV4 genomes are 1.7–2.0 kb [23,24], the full-length genome of PCV5 is 2904 nt. Second, both major open reading frames are longer: the Cap gene spans 1182 nt, about 1.7 times the 645–702 nt of conventional strains, and the Rep gene spans 1056 nt and encodes 351 amino acids (aa), compared with 888–942 nt encoding 296–314 aa in conventional strains [18]; its stem-loop (a 9-nt stem and a 17-nt loop) is also longer than those of conventional types [18]. Third, its sequence divergence is extreme: the PCV5 Rep protein shares at most 26.2% amino acid identity with the four known PCVs (with PCV2), and its Cap protein at most 20.8% (with PCV4), far below the 80% species demarcation threshold of the family *Circoviridae* [18,21]. Fourth, its phylogenetic position lies outside the family: PCV5 clusters outside *Circoviridae* and is most closely related to fur seal feces-associated circular DNA virus (FSfaCV), a 2925-nt circular DNA virus identified in 2013 from the feces of *Arctocephalus forsteri* [25]; at the protein level, twelve PCV5 strains share 85.8–92.6% Rep and 89.1–99.2% Cap amino acid identity with FSfaCV (accession KF246569.1); because these values are amino acid identities, they are not directly comparable to nucleotide-based species demarcation thresholds [18]. On these grounds, PCV5 is best read not as another variant of the known porcine circoviruses but as a CRESS DNA virus from a separate animal lineage that has newly turned up in swine [18].

### 1.3. Scope and Objectives of This Review

The arrival of PCV5 makes this a good moment to step back from strain-by-strain or genotype-by-genotype description and look at porcine circoviruses as an evolutionary problem. We work from a comparative genomics angle. The genomes of PCV1 through PCV5 are laid out side by side first, together with the domain architecture of their Rep and Cap proteins, because we want to understand how the fold of these proteins survives while their sequences drift apart, and what that tension does to taxonomy. From there the review moves on to substitution rates and the dynamics of genetic diversity, with the genotype turnovers that immune selection has produced in PCV2 receiving the closest scrutiny. Host adaptation and cross-species transmission follow, read against host immune pressure, intensive swine production, and global livestock trade. On that basis we argue for an evolutionary history of multiple origins and repeated colonization of swine by unrelated lineages, and we draw out what this implies for viral taxonomy and for One Health surveillance. Two caveats on scope are worth stating at the outset. First, our focus is the two major viral proteins, Rep and Cap; PCV2 also encodes accessory proteins in smaller ORFs, ORF3 among them (implicated in apoptosis) and ORF4 (implicated in innate immune modulation), which mediate pathogenesis but lie outside what we cover here. Second, the review is confined to the porcine circoviruses and to the non-porcine viruses that bear directly on their origins, such as FSfaCV; it is not a survey of the wider CRESS DNA virome.

## 2. Genomic Architecture and Comparative Analysis

### 2.1. Genome Size and Structural Characteristics

**Figure 1:**
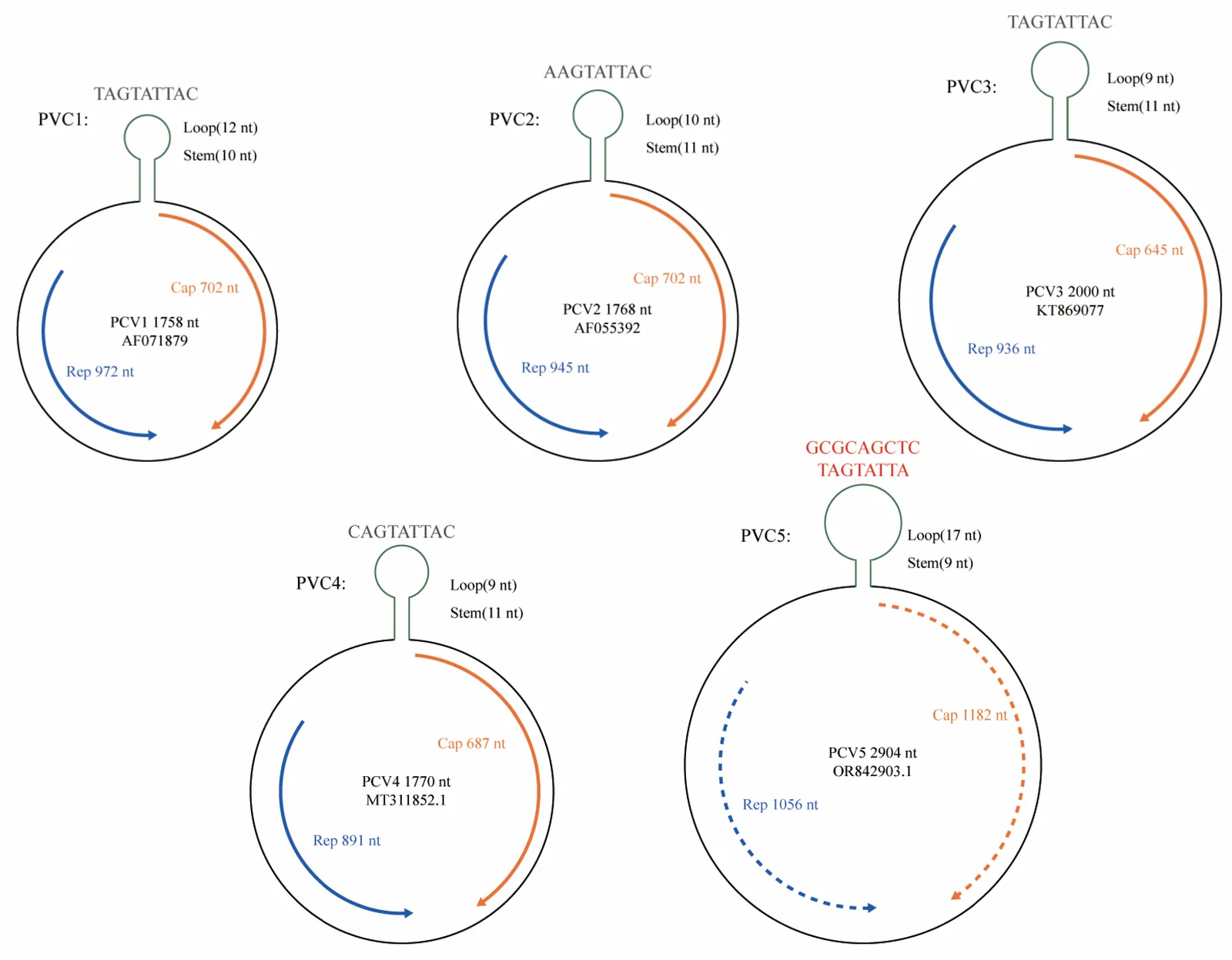
Schematic diagram of the genomic organization of PCV1–PCV5. The conserved canonical nonanucleotide motif at the loop apex of PCV1, PCV2, PCV3 and PCV4 is shown in grey font. PCV5 possesses an elongated loop and lacks this canonical nonanucleotide motif; the nucleotide sequence of its loop is highlighted in red. Solid lines represent sense strands, and dashed lines represent antisense strands, demonstrating the opposite sense/antisense configuration of Rep and Cap genes in PCV5 relative to PCV1–4.

Genome size gives the most direct clue available about the evolutionary trajectory of these viruses. Among the conventional types, PCV1 and PCV2 have the smallest genomes (about 1.76–1.78 kb), PCV3 is about 2.0 kb, and PCV4 is about 1.7 kb [23,24]; PCV5, at 2.9 kb, is the largest of the known PCVs [18] (Figure 1, Table 1). In line with this expansion, the Rep and Cap proteins of PCV5 reach 351 aa and about 394 aa, respectively, compared with 296–314 aa and 214–233 aa in conventional types [18,23]. This simultaneous lengthening suggests that PCV5 may carry regulatory elements or auxiliary functions absent from conventional PCVs, although this hypothesis awaits experimental testing [18]. The polarity of the two major ORFs is also inverted relative to classical PCVs: the PCV5 Cap is encoded on the sense strand and Rep on the antisense strand [19].

The stem-loop in the intergenic region between the two major ORFs is the core cis-acting element that initiates rolling-circle replication: Rep binds to a conserved nonanucleotide motif within this structure and nicks single-stranded DNA to initiate replication [26,27]. Replication also requires the coordinated action of Rep and its truncated isoform Rep′, both encoded by ORF1 [28]. The PCV5 stem-loop has a short stem and a long loop (9 nt and 17 nt) and retains a highly conserved nonanucleotide motif; however, the repeats flanking its origin of replication are more complex than those of conventional types, which may be related to its larger genomic capacity [18].

### 2.2. Comparison of Rep Proteins

Rep is the replicase that initiates rolling-circle replication. Although primary sequences differ widely among strains, their functional architecture is conserved: the canonical three-domain topology consists of an N-terminal HUH superfamily endonuclease domain containing RCR catalytic motifs, a central oligomerization domain, and a C-terminal superfamily 3 (SF3) helicase/ATPase domain containing Walker A/B motifs [18,26]. Certain catalytic residues, the tyrosine of RCR motif III among them, are strictly conserved in every strain; the biochemistry of rolling-circle replication has evidently not changed over their long evolutionary history [26]. For PCV5, structural predictions and experimental data show that its Rep retains a global fold highly similar to that of PCV2 Rep; a truncated variant (Rep NΔ118) expressed in soluble form in *Escherichia coli* retains ATPase activity and forms oligomers [18]. Sequence divergence on this scale alongside a shared fold points to strong purifying selection on Rep: whatever drift accumulates in the sequence, the conformation required to recognize the origin of replication must be preserved [26].

Sequence divergence among the conventional genotypes is comparatively modest: PCV1 and PCV2 are each other’s closest relatives—the highest whole-genome identity of any pair among the five, and a single clade in every phylogeny inferred to date—whereas PCV3 and PCV4 share only about 40–60% identity with the PCV1/PCV2 pair [23,24]. PCV5 is the clear exception, sharing at most 26.2% Rep amino acid identity with swine-origin PCV1–PCV4 but 85.8–92.6% amino acid identity with FSfaCV [18]. Protein-level identity of this kind cannot be measured directly against the 80% whole-genome nucleotide threshold used to demarcate species in the family *Circoviridae* [21,22]; rather, it is the concordance of deep protein divergence with whole-genome phylogeny that places the distance between PCV5 and the known PCVs beyond both the species and the genus boundaries of that family, and PCV5 is most plausibly congeneric with FSfaCV within a lineage that awaits formal taxonomic definition. Its formal status awaits official demarcation by the ICTV; a genome-wide comparative analysis published in 2026 proposed classifying it in the genus *Macochavirus*, as a swine-associated lineage designated PMV [20].

### 2.3. Comparison of Cap Proteins

Cap is the sole structural protein, encoded by ORF2. It mediates attachment, entry into host cells, and genome packaging, and carries the dominant B-cell and T-cell epitopes, making it the main target of immune recognition and neutralizing antibodies [23,29]. Cap evolves faster than Rep, and its sequence is therefore the primary marker for PCV genotyping [29,30].

The Cap proteins of PCV1–PCV4 are 214–233 aa long, whereas the PCV5 Cap gene spans 1182 nt and encodes a protein of about 394 aa, roughly 1.6 times the size of conventional capsids [18]. PCV5 Cap shares at most 20.8% amino acid identity (with PCV4) with the four conventional PCVs—less than its Rep protein (26.2%)—and is the most divergent capsid among circovirus types [18]. Such low identity implies a substantial antigenic distance from conventional porcine circoviruses, and it is unlikely that existing PCV2 vaccines confer effective cross-protection against PCV5 [18,23].

In domain organization (Table 2), the N-termini of Cap proteins of all types are arginine-rich and carry a conserved nuclear localization signal (NLS) that directs the capsid protein to the nucleus for viral genome replication and assembly [31,32]. Antigenic epitopes lie mainly in surface-exposed loops: neutralizing epitopes of PCV1 and PCV2 are concentrated in loops CD and HI [33,34], and PCV2 has additionally evolved an immunodominant C-terminal decoy epitope [35]. PCV3 shows a more variable epitope distribution, and epitopes in PCV4 are more dispersed [29,36]. No epitope mapping has yet been reported for PCV5 Cap; whether it has evolved an immune-evasion structure similar to the PCV2 decoy epitope is a key question for future antigenic screening and immunological work.

### 2.4. Revisiting Phylogenetic Relationships

**Figure 2:**
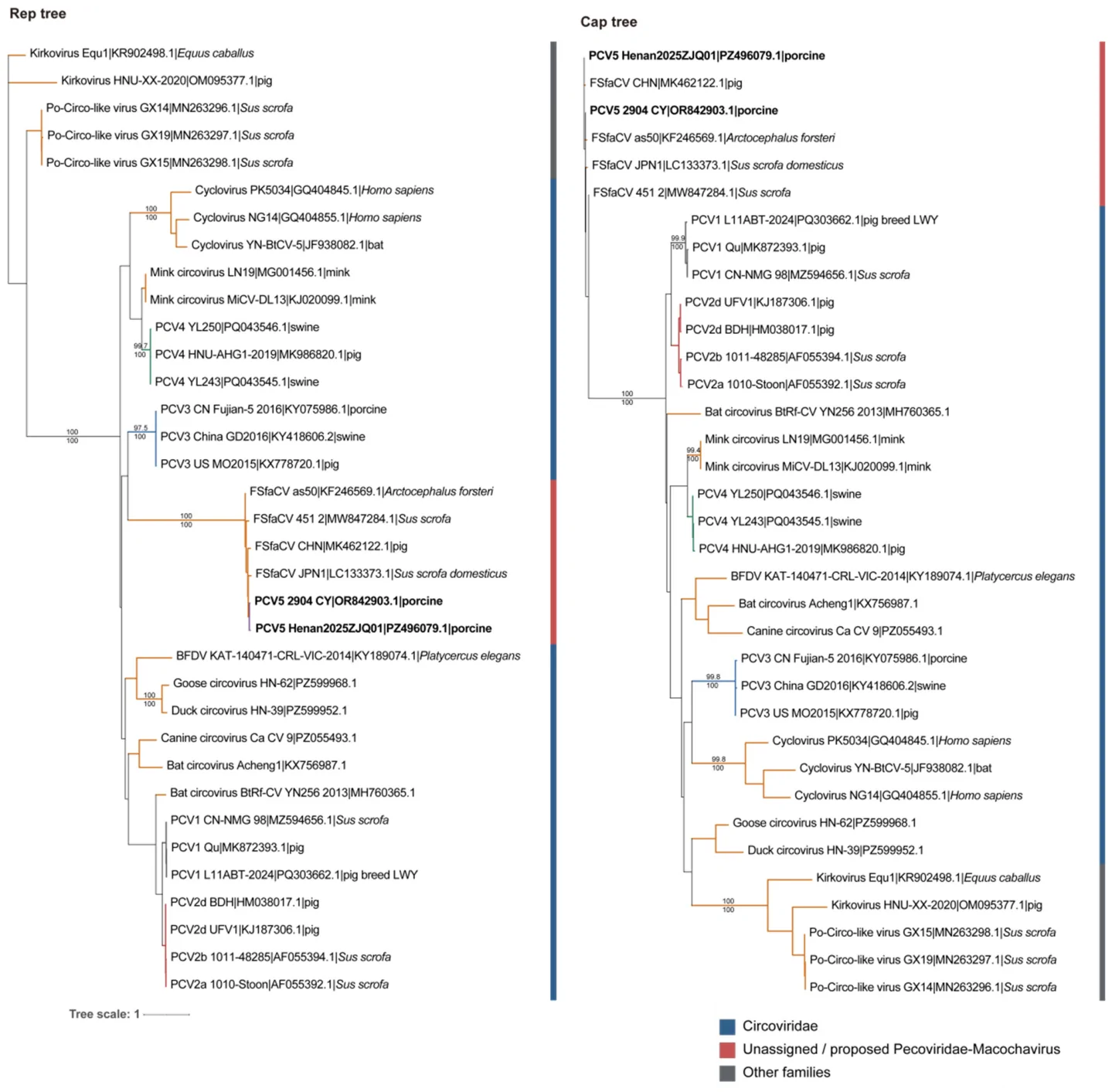
Phylogenetic tree of PCVs based on the amino acid sequences of Rep and Cap.

Before the discovery of PCV5, phylogenetic trees based on Rep and Cap sequences had a stable overall structure: PCV1 and PCV2 formed a tightly clustered clade, while PCV3 and PCV4 each occupied separate lineages [23,37]. PCV5 has upset this picture: instead of joining the swine-origin circoviruses, it forms a well-supported clade with CRESS DNA viruses such as FSfaCV, clearly outside the family *Circoviridae* [18] (Figure 2), and a genome-wide analysis in 2026 proposed reclassifying it into the genus *Macochavirus* of the family *Pecoviridae*, as the porcine-associated lineage PMV [20]. Strictly speaking, within the family–genus–species framework, PCV5 would not belong to *Circoviridae* at all. This suggests that the porcine circoviruses are a polyphyletic assemblage assembled from several distinct sources through recurrent cross-species colonization, rather than a single monophyletic clade [18,24].

The placement of PCV5 has consequences for CRESS DNA virus classification that reach past this one species. Metagenomics adds to the recognized diversity and host range of these viruses year by year [38], and the framework doing the sorting leans on Rep phylogeny and structure at higher ranks while demarcating species within the family *Circoviridae* by 80% whole-genome identity [21,22,26]. Neither half of that arrangement survives contact with PCV5. Rep can stay strongly conserved while Cap diverges antigenically beyond recognition, and Cap divergence taken on its own can inflate the apparent distance between two viruses. Any placement of PCV5 and its relatives that is to hold up will have to weigh Rep, Cap, and whole-genome evidence simultaneously, which is what a 2026 analysis of FSfaCV/PCV5-related viruses did when it resolved PCV5 as a distinct porcine-associated lineage inside the genus *Macochavirus* [20].

## 3. Evolutionary Rates and Genetic Diversity

Among animal circoviruses, the porcine viruses have the longest longitudinal sequence record, but that record is very unevenly distributed across the five species. This section does two things. It establishes the substitution-rate baseline against which porcine circoviruses should be read, and it takes stock of the diversity described for each of PCV1 to PCV5. Where the depth of coverage differs between viruses, the reason is the state of the evidence rather than the emphasis of this review, and we say so rather than fill the gaps by inference.

### 3.1. One of the Fastest-Evolving DNA Viruses

Because DNA polymerases proofread, DNA viruses have long been considered genomically stable, and PCV2 was an early exception to this view. Bayesian molecular clock analyses estimated its whole-genome substitution rate at approximately 1.2 × 10^−3^ substitutions per site per year (95% HPD: 8.2 × 10^−4^–1.6 × 10^−3^), far above the rates of typical double-stranded DNA viruses [39]. At the time, this was among the highest substitution rates ever estimated for a DNA virus, approaching the evolutionary dynamics of small RNA viruses [39].

Rate estimates should be compared with care, because they depend strongly on the timescale of observation, and short-term estimates systematically exceed long-term ones. Even so, PCV2 remains an outlier. Among other single-stranded DNA viruses, canine parvovirus evolves at approximately 1.7 × 10^−4^ substitutions per site per year [40] and *tomato yellow leaf curl virus* at about 2.88 × 10^−4^ [41], both 3- to 7-fold lower than PCV2. The gap widens for double-stranded DNA viruses: variola virus evolves at roughly 8.5 × 10^−6^ [42] and herpes simplex virus 1 (HSV-1) at 1.82 × 10^−8^ substitutions per site per year [43]. Even hepatitis B virus (HBV), which replicates through reverse transcription and is often described as RNA-like in its evolution, shows short-term rates mostly on the order of 10^−5^–10^−4^ substitutions per site per year, declining as the timescale lengthens [44] and remaining consistently below that of PCV2 [39].

Why does PCV2 evolve at such an RNA-like pace? Several mechanisms probably act together. The genome is extremely compact yet dense with functional sites, replication turnover is exceptionally high, and recombination occurs frequently during rolling-circle replication [39,45]. Evolution at this speed supplies variation faster than immune-driven selection can clear it. What that implies for genotype turnover is taken up in Section 6.

### 3.2. Genotypic Evolution of PCV2

Canada reported the first cases of postweaning multisystemic wasting syndrome in the early 1990s, and by the late 1990s PCV2 had been identified as the cause [6,46]. The record since then is one of repeated global genotype shifts [7,39]. Before 2000, PCV2a predominated. Around 2003, PCV2b took over worldwide, at the same time as PMWS was occurring on a wide scale [47,48]. PCV2d has been gaining ground since 2013 and is now the principal lineage in most regions [30,49,50] (Table 3). Nomenclature was messy in the early years, with several competing schemes built on whole-genome or Cap-based clustering [48,51]. That ended in 2018, when Franzo and Segalés put forward a unified ORF2-based framework: clusters bounded by a maximum intra-cluster *p*-distance of 13%, bootstrap support above 70%, and no fewer than 15 sequences per cluster. Under their framework eight genotypes are recognized, PCV2a through PCV2h, and the scheme has since become the working standard [52].

Several motifs distinguish PCV2d, and all of them sit where immune selection would be expected to leave traces on the capsid surface. SNPLSV occupies capsid residues 86 to 91 within the CD loop, TGID lies within the GH to HI loops, and PLNPK spans residues 230 to 234 near the C-terminus. Positively selected sites cluster in the immune recognition regions, A68N in IRR-A among them, G119 and V130 in IRR-B, and L167, T190, D194 and F202 in IRR-C [45]. Vietnamese surveillance data show what followed in unusually sharp relief. The dominant genotype there changed from PCV2b to PCV2h around 2008, PCV2d began climbing after 2014, and by 2022 and 2023 PCV2d made up 80.9% of detected strains against 19.2% for PCV2b [53]. Worth noting as well is that PCV2h is a product of inter-genotypic recombination involving PCV2a, PCV2b, and PCV2d, and that its sequences tracked closely with vaccine strains then in local use in Vietnam [45]. Documented instances of vaccine-related recombination in the field are few, so this case carries real weight.

The selection pressures that drive this turnover are considered in Section 6.2 and Section 6.3.

### 3.3. Genetic Diversity of PCV3, PCV4, and PCV5

Where PCV3 came from, and how it evolves, have been argued over ever since the virus was identified in 2016, and the argument has yet to settle. Roughly 55% Rep identity separates it from known swine-origin circoviruses, and it sits nearer to bat circoviruses [8]. On that basis most authors favor an entry into swine by spillover from bats or some other wild animal [12]. Once established, the virus expanded globally at speed. A Chinese survey in 2017 detected PCV3 on 35 farms in 11 provinces, at 68.6% farm-level positivity (24/35) and 34.7% sample-level positivity (77/222), frequently together with PCV2 [10]; later nationwide surveillance bore out high prevalence across most provinces of the country [12,13].

PCV4 presents a different picture again, and a more divergent one: it shares just 43.2% to 51.5% whole-genome identity with PCV1 to PCV3 [14,54]. South Korea was the next to report the virus, in pooled tissue samples from six of nine provinces (3.28% positivity) [15], with individual swine positivity reaching 39.3% [16]; Thailand followed, where only 3 of 734 samples were positive, and all three were co-infections with PCV2 or PRRSV [17]. Dissemination along Asian swine trade networks would account for this distribution well. The label “unresolved” can be stated more precisely: the 43.2% to 51.5% whole-genome identity of PCV4 to PCV1 to PCV3 places the virus outside each of their lineages, yet no intermediate or progenitor genotype has been recovered from any host, and sampling of hosts other than domestic pigs remains too sparse to test whether a closer relative circulates elsewhere; until such a relative is found, neither an independent origin nor membership in a known lineage can be established [14,54].

Within PCV5, diversity remains narrow. Rep identities across the 12 known strains span 87.7% to 99.7% and Cap identities span 89.1% to 99.2%, with Cap the more variable of the two proteins [18]; it is therefore on the capsid that immune pressure would be expected to register first. The Henan strain isolated in 2025, PCV5-Henan2025-ZJQ01 (2903 nt), shares 96.7% whole-genome nucleotide identity with the prototype, together with 96.9% Cap and 96% Rep identity at the amino acid level [19]. Differentiation is already under way as the virus spreads geographically.

It is tempting to read this against PCV2, where a phase of limited diversity was followed by successive genotype replacements [37,39]. We think the comparison is worth drawing but should not be pressed, and our own account of PCV5’s origins argues against pressing it. PCV5 did not emerge by stepwise divergence within swine: it falls outside the family Circoviridae, and its closest relatives are CRESS DNA viruses, so its appearance in pigs reads as a single cross-taxonomic host jump (Section 2.4 and Section 6.1) [18,20]. The record for PCV2 is instead one of within-swine lineage turnover under immune pressure, and part of that turnover—the PCV2a to PCV2b replacement—predates the widespread use of commercial vaccines and remains unexplained (Section 6.2). A trajectory assembled by one host jump need not resemble one assembled by two decades of within-host selection, and the vaccine-driven pressure that dominates PCV2’s later record does not yet exist for PCV5.

What can be said with confidence is therefore narrower than a prediction. If Cap diversity in PCV5 does expand, the earliest phase of that process is the one represented by the 12 sequences available today, and it will not be recoverable afterwards. Prospective sequencing is justified on those grounds alone, whether or not the analogy with PCV2 holds.

## 4. Host Adaptation Mechanisms

### 4.1. Selection Pressures Driven by Host Environments

Host environment exerts a strong influence on the pace of PCV evolution. *Wild boars* populations live at low density, hold diverse genetic backgrounds, and receive no vaccination, so the viral evolution within them comes closer to neutral drift than to anything else. *Domestic pigs* stand at the opposite end of the spectrum. Immune-mediated selection presses on the exposed surface loops of Cap, and non-synonymous substitution rates run significantly higher in domestic-derived than in wild boar-derived strains [55,56]. Evolution accelerates further under high stocking density and frequent passage, and mass vaccination stacks a directional force on top of everything else [50]. The chronology deserves a closer look. Commercial vaccines were not yet in wide use when the first genotype replacement, PCV2a to PCV2b, took place around 2003, so fitness differences among viruses during the expanding PMWS epizootic are the more credible explanation [47,48]. PCV2d rose later, in step with mass vaccination. Clinically the monovalent PCV2a-based vaccines performed well, yet a selection window favoring this heterologous genotype may have been an unintended consequence of their use [30,50].

Receptor usage forms the other half of host adaptation. Glycosaminoglycans, heparan sulfate (HS) in particular, serve as the principal attachment factors for PCV2 [57], and HS binding occurs through multiple low-affinity sites distributed across the capsid surface [58]. A constraint follows from this arrangement. Antigenic epitopes can be reshaped in the effort to escape neutralizing antibodies, but the charge and conformation of receptor-binding sites must be preserved at the same time, and monocyte entry efficiency tracks the amino acid properties of the capsid surface directly [59]. Immune evasion and receptor binding thus pull against each other, and the trade-off between them lies at the center of PCV host adaptation [58,59]. Applied to PCV5, the question that comes first is whether its extended Cap carries new receptor-binding modes, a broader tissue tropism, or genuine cross-species potential [18].

### 4.2. Cap Protein Mutations and Host Adaptability

Beyond antigenicity, amino acid substitutions in Cap can influence how efficiently the virus infects. When 1682 Cap sequences were analyzed together, the high-frequency PCV2 mutations proved to concentrate on the capsid surface and to accumulate positively charged polar amino acids preferentially. The effect works in two directions, because a surface electrostatic potential shifted in this manner binds negatively charged cellular receptors more strongly, while the very same substitutions modify epitope antigenicity and bury epitopes deeper beyond the reach of antibodies. Receptor affinity and immune escape thus improve together, in a single step [60]. Most of the control over receptor binding and viral entry sits with the C-terminus of Cap and with certain surface loops, BC and GH in particular [61].

A second core strategy of host adaptation is epitope remodeling, which the capsid surface permits because it carries several conformational neutralizing epitopes. The immunologically important neutralizing epitopes of PCV2 fall mostly within Cap residues 50 to 90, 110 to 150, and 160 to 200 [33,62]. Replacement of one or a few key residues seldom disturbs the overall fold. What it can do, through steric hindrance or charge mismatch, is shift local conformation by just enough to defeat existing neutralizing antibodies [34,62]. Once mutations of this kind have accumulated within a viral population, cross-reactivity with the immunity primed by earlier strains begins to decline. This is the process that drove the global lineage transition of PCV2 from 2a to 2d at the molecular level [30,50]. Innate immunity is circumvented as well. PCV interferes with the cGAS–STING–type I interferon pathway and modulates host regulated cell death, and both activities help it persist while dysregulating host immunity [63].

Vaccine-based control operates under permanent strain from antigenic drift that never pauses. PCV2a or PCV2b backbones underlie most commercial vaccines [30], whereas PCV2d now dominates field strains, and the antigenic match has been slipping accordingly. Epidemiological studies have already documented the consequences, in the form of breakthrough infections and weakened cross-protection [50]. Current vaccines fall short of sterilizing immunity, and the field has converged on the need for multivalent vaccines covering diverse genotypes, together with formulations updated as new dominant strains emerge [30,50,64]. Further down the pipeline sit recombinant subunit vaccines, virus-like particles (VLPs), and DNA vaccines, some designed to be multivalent or modular against genotypic diversity and co-infection [63].

### 4.3. Structural Conservation of the Rep Protein as the Precondition for Cross-Host Function

Rep sits at the other pole from the hypervariable Cap: structurally, it is stable. PCV5 Rep shares extremely little amino acid identity with the Reps of other CRESS DNA viruses, yet structural predictions and biochemical assays both confirm that it keeps the classic three-domain fold, with the catalytic centers (the HUH and Walker motifs) strictly conserved [18,26]. Divergence on that scale alongside a conserved fold is hard to explain by gradual change alone; it fits better with the idea that CRESS DNA virus lineages assemble new Rep/Cap combinations through recombination, which they probably do often [26]. PCV5 Rep also recognizes its native stem-loop specifically and initiates rolling-circle replication in vitro [18]. The module still works, in other words, after crossing a host species barrier.

More broadly, the structural and evolutionary characteristics of PCV5 Rep point to a large, undersampled pool of CRESS DNA viruses in nature whose structural diversity exceeds what is currently known; PCV5 may be only the visible fraction of this pool that has reached swine [18,26]. It therefore serves as a bridge between the environmental and wildlife reservoirs of CRESS DNA viruses and domestic livestock, and its study may clarify how such viruses transition into livestock pathogens. Within the argument of this section, Rep conservation is not incidental to host adaptation but its precondition: a replication module that keeps working across host boundaries is what lets a CRESS DNA virus survive the crossing before any adaptation to the new host has begun.

## 5. Cross-Species Transmission Potential

### 5.1. Expansion of the Host Range

**Figure 3:**
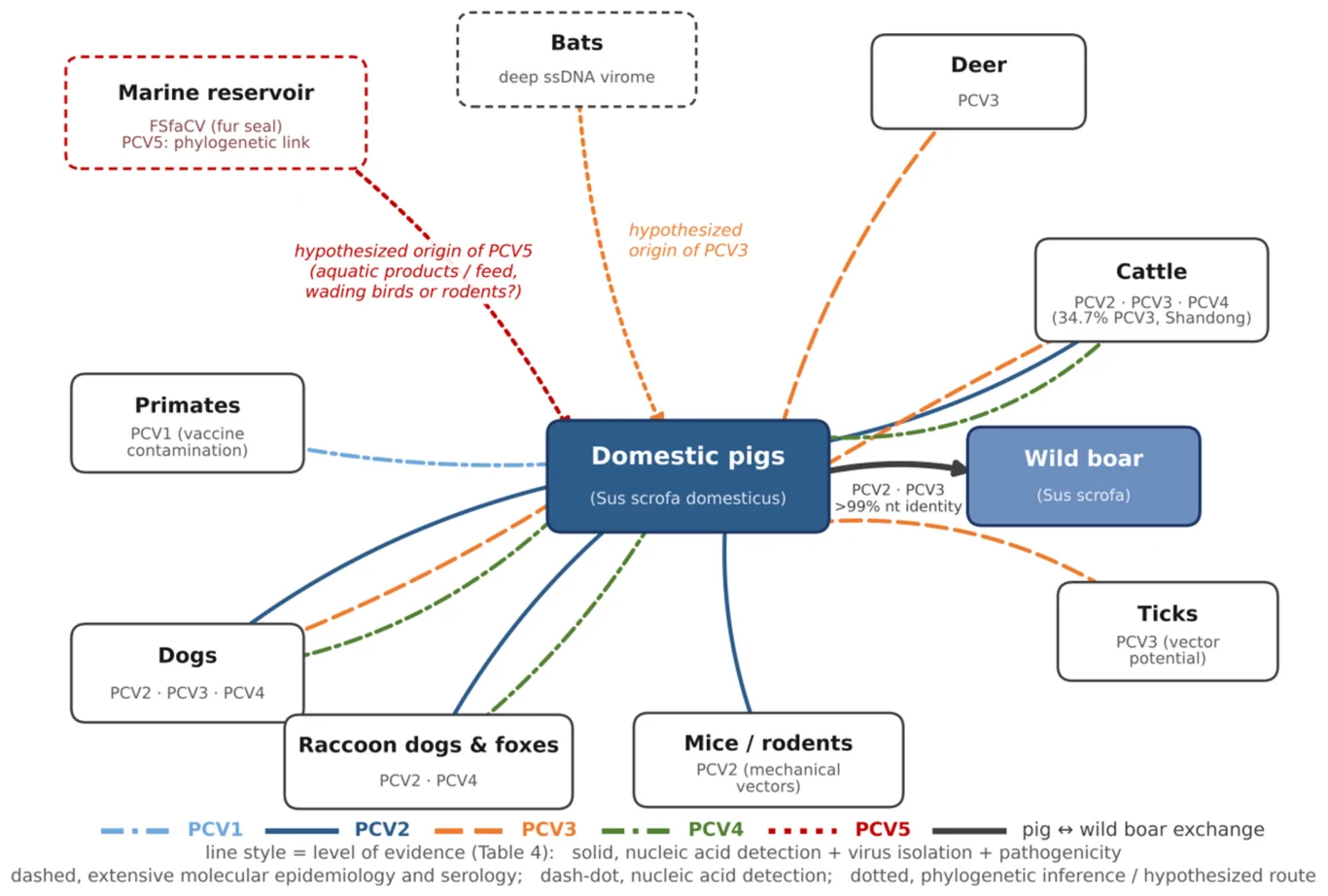
Cross-host transmission of porcine circoviruses shown as a network diagram. Line style indicates the highest grade of evidence currently available for each link, as graded in Table 4: solid, nucleic acid detection plus virus isolation and pathogenicity; dashed, extensive molecular epidemiology and serology; dash-dot, nucleic acid detection; dotted, phylogenetic inference or a hypothesized route. The network reflects the evidence published to date and should be read as a snapshot of a still-moving-fast story: the host range of porcine circoviruses continues to expand with ongoing surveillance, and a link is upgraded only when targeted isolation or experimental infection studies are carried out, not by the passage of time alone.

High-throughput sequencing and broad-spectrum PCR primers have steadily widened the known host range of PCVs, making cross-species spillover a growing concern in veterinary virology [24] (Figure 3). The natural host spectrum of the family *Circoviridae* extends well beyond the genus *Sus scrofa*: metagenomic studies have detected highly diverse circoviruses in various domestic animals, bats, and even wild chimpanzees and human feces [38,65,66]. PCV3 has been detected in a wide variety of non-porcine hosts, including cattle, dogs, and deer. In Shandong Province, the nucleic acid positivity rate of PCV3 in cattle herds reached 34.7% (74/213) [67], markedly higher than PCV2 detection levels in ruminants, and prevalence was higher in cattle with respiratory signs, suggesting an association with bovine respiratory disease complex (BRDC) [67]; natural PCV3 infection in dogs has also been documented in several countries [68]. Nor is PCV2 confined to the genus *Suss crofa*. Detection and disease reports exist for raccoon dogs, foxes, and dogs [69], and for mice as well [70]. Strains carried by rodents living on swine farms are highly similar to those from co-located pigs, which fits a role for these animals as mechanical vectors or intermediate hosts [70]. Ticks have yielded PCV nucleic acids too [71]. No one has yet demonstrated viral replication in arthropods, but vector potential of that kind is not something that can simply be dismissed.

Not all of these observations carry equal weight. What is known about PCV1 comes mainly from cell culture contaminants and vaccine contamination events. PCV2, by contrast, has actually been isolated from several non-swine species, and its pathogenicity has been demonstrated. The evidence for PCV3 and PCV4 currently amounts to nucleic acid detection plus serology in multiple species, and no more. As for PCV5, host evidence is restricted to swine, and any talk of cross-species potential rests on phylogenetic inference alone [18]. A disciplined framework for judging the host range of newly discovered circoviruses can be built by grading evidence in exactly this manner, from nucleic acid detection upward through virus isolation, experimental infection, and demonstrated pathogenicity (Table 4).

Two different situations lie behind the lower evidence grades, and they should not be conflated. The primary studies behind most detection-only links were designed as surveillance, their endpoint being nucleic acid detection rather than isolation or pathogenicity [67,68,69,71]; in those hosts, higher-grade evidence has been neither achieved nor reported as attempted. Isolation itself, moreover, is not simply a matter of time: PCV3 has proved difficult to culture, isolates remain scarce, and the few available come from porcine samples [72]. The solid lines for PCV2, by contrast, were earned by decades of targeted work, including the experimental demonstration of replication and transmission in rodents [70]. Upgrading a link, in short, requires such studies; surveillance alone adds hosts, not evidence.

### 5.2. Spillover Between Domestic Pigs and Wild Boars

PCV circulation and genetic diversity are sustained in part by the exchange that goes on between *domestic pigs* and *wild boars*. Surveys in Portugal, Italy, Poland, and other European countries have recorded high rates of both PCV2 and PCV3 infection in *wild boars* populations [55,56,73]. In the same regions, viruses from *wild boars* and from *domestic pigs* often intermingle on phylogenetic trees and frequently share more than 99% nucleotide identity [55,56,73]. Free-range farms overlapping *wild boars* habitat create the riskiest version of this interface, since *wild boars* can act as reservoir hosts for viruses such as PCV3 and pass them back to commercial pig farms [55,73]. What these surveys establish, however, is an active exchange interface—phylogenetic intermingling and near-identical genomes on both sides—not the direction of any single event; prevalence data alone cannot assign donor and recipient roles, and formal gene-flow analyses across this interface are still lacking.

Host species barriers can also be crossed by recombination. In PCV2 the process is remarkably active, and its hotspots cluster around the Rep/Cap gene junction [73,74]. What it can achieve is illustrated by PCV2h, a recombinant of PCV2a, PCV2b, and PCV2d whose epidemiology ties it to vaccine use in Vietnam (Section 3.2). Swapping a Rep/Cap module changes receptor-binding and antigenic properties within a short time, while an already proven replication backbone is retained. Modular architecture of that sort extends the adaptive reach of the virus across *domestic pigs*, *wild boars*, and other mammalian hosts [26,45,74].

### 5.3. Evolutionary Origin and Phylogenetic Insights

A spillover from bats is at present the best-supported origin hypothesis for PCV3. Phylogenies built from whole genomes place the virus close to bat circovirus lineages, and strong signals of positive selection mark its capsid gene, the type of signature expected to arise during a host jump [8,12]. Circular ssDNA viruses occur in bats with exceptional diversity, forming a substantial natural reservoir for events of this kind [75]. Ecological modeling contributes a further observation, namely that bat habitats and swine facilities have overlapped for a long time, and an overlap of long standing supplies exactly the contact a spillover would require [12].

PCV5 is most closely related to FSfaCV [18], and this affinity has been read as evidence of a host jump out of a marine reservoir. That reading should be treated as a testable hypothesis rather than a conclusion. FSfaCV is known only from fecal metagenomes of New Zealand fur seals [25], and a virus detected in feces need not be a virus of the animal that produced them: it may equally derive from prey, from the environment, or from another animal in the vicinity of the seal. Nor is the direction of any marine–terrestrial exchange established. FSfaCV-like viruses had already been detected in swine feces in Japan by 2017 [76], years before PCV5 was named, a finding difficult to reconcile with a simple seal-to-pig spillover and one that leaves open the possibility of a shared, still unsampled source. If a marine–terrestrial interface is involved, the intermediate host, the route of transmission, and the natural reservoir all remain unknown. The candidate pathways that surveillance could test are concrete: marine-derived feed ingredients and aquatic by-products; the movement of animals, vehicles, equipment, and personnel; synanthropic rodents and wild birds; wastewater and sewage; slaughterhouses; and the live-pig trade. Framing the question this way keeps the full ecological interface in view instead of presuming a marine origin at the outset.

Pieced together, the evidence points to multiple origins and repeated cross-species colonization of swine. PCV1 and PCV2 form one clade, long adapted to the genus *Sus scrofa* (read here as closest known relatives within swine, pending a formal coalescent reconstruction); PCV3 most plausibly reflects an independent cross-species spillover, though the bat lineage concerned has not been pinned to a particular species or geography [12]; the origin of PCV4 remains unresolved for the reasons set out in Section 3.3 [14,54]; and PCV5 clusters with a seal feces-associated CRESS DNA virus, a relationship compatible with an origin at the marine–terrestrial interface but not yet demonstrative of one [18]. Porcine circoviruses, in other words, are not the product of progressive intra-host evolution of a single ancestor, but the accumulated record of discrete spillover events that converged on one host species. This conclusion has a practical implication: rather than waiting passively for a PCV6 to appear, surveillance should actively mine the vast CRESS DNA virome at the wildlife–environment–livestock interface [18,24].

## 6. Evolutionary Drivers

**Figure 4:**
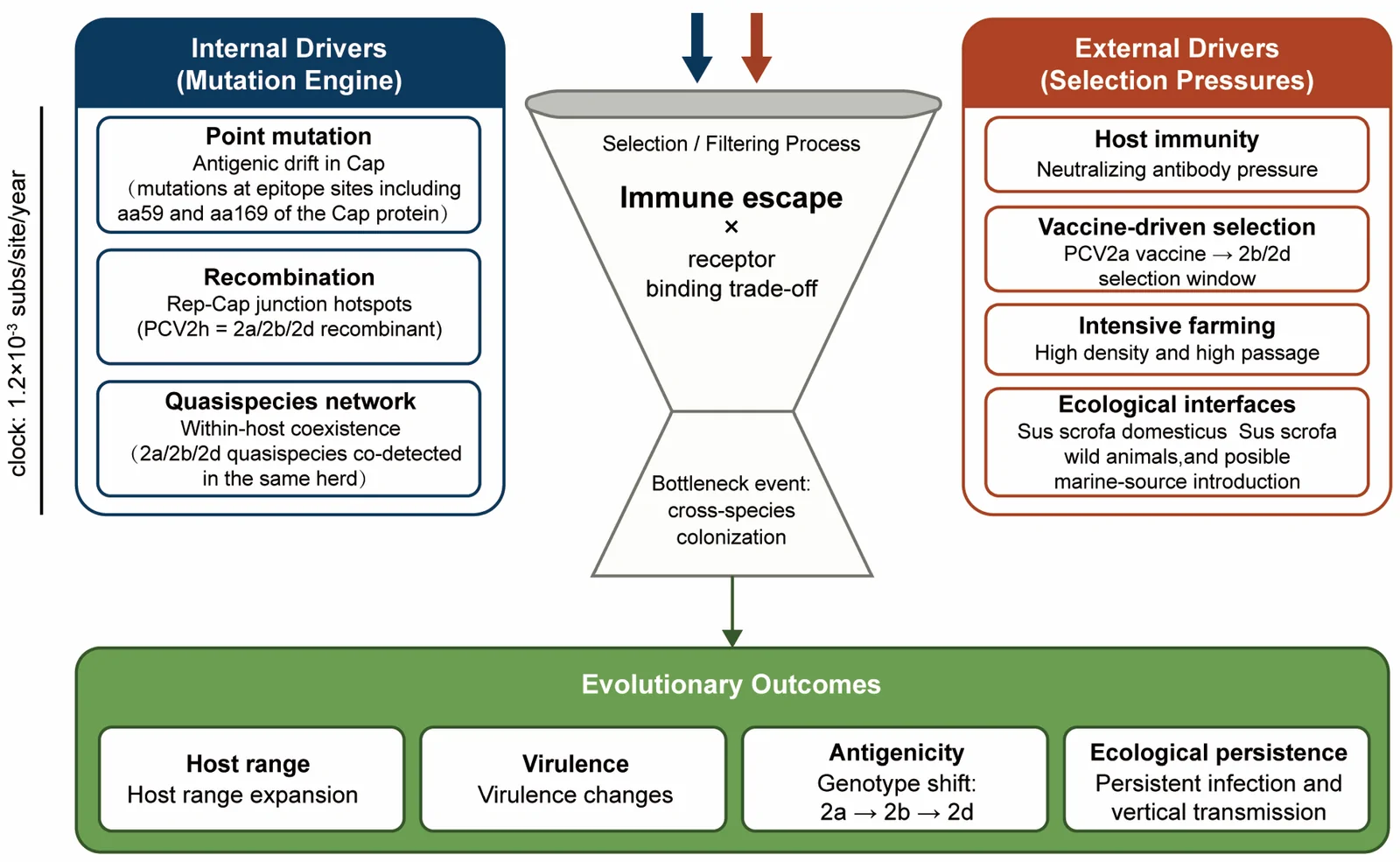
Conceptual diagram of the factors driving PCV evolution.

The evolution of porcine circoviruses can be viewed as a funnel (Figure 4). Intrinsic genetic processes—above all point mutation and recombination—continuously generate genetic diversity, while extrinsic pressures such as host immunity, intensive farming, and ecological contact filter it. The interaction between the two forces decides the outcomes that matter for animal health, from the widening or narrowing of host range, to the direction virulence takes, to whether the virus manages to persist in the ecosystem.

### 6.1. Recombination and Point Mutation

Each genotype mixes mutation and recombination in its own proportions [24,74]. Both processes are prominent in PCV2. Quasispecies networks of considerable complexity develop inside a single host, and longitudinal sampling has documented genetic variation accumulating under host immune selection; co-mingling of pigs from different origins then sends co-infection and recombination rates up sharply [77]. Recombination hotspots are scattered through the genome, above all at the Rep–Cap junction [74], and their presence is largely what makes inter-genotypic recombination so common, as the PCV2h example in Section 3.2 illustrates. Antigenic drift is kept running at the same time by positive selection on Cap, strongest in the decoy epitopes and receptor-binding regions [35,60,62]. PCV3 takes the opposite path, its evolution proceeding mainly by point mutation, with little sign of inter-genotypic recombination [12], a pattern consistent with, though not demonstrative of, a relatively settled state of adaptation to swine and one that awaits confirmation by formal recombination screens and substitution-rate estimates for PCV3. As for PCV5, no significant recombination has been detected in it so far [18], and its emergence reads less like stepwise evolution within swine than like one cross-taxonomic host jump.

### 6.2. Host Immune Pressure and Vaccine-Driven Selection

Within intensive swine production, vaccination holds a peculiar position. It is the principal instrument for controlling PCV2-associated disease [78], and at the same time one of the strongest directional forces acting on the evolution of the virus [50]. Most of the positively selected sites in PCV2 lie on the surface-exposed regions of Cap [60], and antibody binding affinity can be cut substantially by non-synonymous mutations inside antigenic epitopes [62]. Why PCV2a yielded to PCV2b remains an open question, given that the replacement predates the widespread use of commercial vaccines. PCV2d, which emerged afterwards, is the case where vaccine-driven selection bears more directly—though the claim rests on temporal coincidence and on the immunological gap described next, not on a measured selection coefficient; dedicated dN/dS and antigenic-site comparisons across the PCV2a-to-PCV2d transition are the missing molecular evidence, and the timing correspondence between the rise of PCV2d and vaccine coverage deserves the same scrutiny given above to the unexplained PCV2a-to-PCV2b replacement. Homotypic protection against PCV2a is where today’s vaccines perform best, whereas cross-protection against PCV2d stays incomplete, and that immunological gap plausibly assisted the colonization and spread of PCV2d [30,50]. Formally the process resembles antigenic drift in influenza, and it accounts for the decline of vaccine efficacy over time.

### 6.3. Farming Systems and Global Trade

Forces acting at a much larger scale shape the phylogeography of porcine circoviruses. Oronasal transmission, the fecal–oral route, and vertical transmission all operate in PCV2, and because the majority of infections remain subclinical [79], infected animals frequently travel without being detected. Bayesian phylogeographic analyses have followed PCV2 across continents along global live-swine trade routes, and what emerges as the chief vehicle of global transmission is the movement of asymptomatic animals [39,80]. China holds the world’s largest swine population and occupies the center of the network, functioning at once as a major source and as a hub for PCV2 and PCV3 [37,81]. An effective population base of that magnitude sustains high genetic diversity, and source–sink dynamics follow, with dominant genotypes such as PCV2d flowing outward along the live-pig trade to neighboring regions and beyond [81]. Vaccination strategy and biosecurity also vary from one trading region to the next, so the selective environment is heterogeneous. Lineages imported into a region adapt locally and are then re-exported, and the sequence of importation, selection, adaptation, and re-export repeats itself [79].

Tempo of viral replication is set by stocking density and management as well. Passage comes frequently on high-density farms, and mutations get little time to accumulate between rounds; low-density settings offer the virus fewer chances to move between herds, and evolution slows down accordingly. Specific pathogen free (SPF) farms and other high-biosecurity environments keep external introductions out effectively, yet a risk peculiar to them remains. Should an outbreak occur inside a restricted host population, the virus passes through a severe genetic bottleneck, and bottleneck events of that kind are capable of forcing abrupt adaptive change. Surveillance design and risk modeling would therefore do well to recognize that the relationship between biosecurity level and evolutionary rate is not a linear one.

## 7. Perspectives and Conclusions

### 7.1. Future Research Directions

The molecular epidemiology of porcine circoviruses has been studied extensively, yet knowledge of PCV5 and of the wider CRESS DNA virus ecological network is still rudimentary [18,24]. Even for PCV2, the most thoroughly studied genotype, several obstacles to moving from disease control to pathogen eradication—vaccines that do not fully prevent infection and shedding, the difficulty of establishing etiology by routine diagnostics, and the lack of standardized viral load and serological thresholds—have only recently been delineated systematically [64]. Reports on PCV5 are so far largely limited to epidemiological surveillance and genomic characterization [18]. PCV5 DNA has been detected by PCR in the heart, liver, spleen, lungs, kidneys, brain, and intestines of affected pigs, and viral load correlates positively with clinical disease severity [18]. These observations warrant restraint in interpretation: PCR detection shows that viral DNA was present in a tissue, not that the organ supports viral replication, and detection in the brain does not by itself establish that the virus crosses the blood–brain barrier. Demonstrating true tissue tropism will require in situ hybridization or immunohistochemistry co-localizing viral nucleic acid or antigen with lesions, together with quantitative viral-load and pathology scores from prospective case–control studies. Koch’s postulates remain unfulfilled for PCV5: whether monoinfection reproduces the clinical lesions, and how much PCV5 contributes to porcine circovirus-associated disease, must be established in experimental infection studies, ideally beginning with an infectious clone and a porcine challenge model; mouse models have proven unsuitable, underlining the need for porcine infection models and standardized reverse genetics systems [18]. The permissive cell types and entry receptors of PCV5 are unknown, although newly established in vitro cell culture systems and the successful purification of ~20 nm viral particles provide a starting point for mechanistic work [18]. Finally, while bioinformatic evidence indicates that PCV5 replicates by rolling-circle replication [18,26], its specific regulatory elements remain uncharacterized.

Cross-species risk assessment should be a research priority. Given the phylogenetic proximity of PCV5 and FSfaCV, surveillance for CRESS DNA viruses in marine–terrestrial transitional ecosystems should be intensified, together with serological and molecular monitoring of wildlife, companion animals, and rodents surrounding swine farms to quantify the risk of PCV5 colonization in non-swine hosts [18]. Methodologically, targeted PCR cannot capture highly divergent novel viruses, because primer design lags behind discovery. Unbiased metagenomic virome screening of unexplained respiratory, diarrheal, and reproductive disease—coupled with routine surveillance at slaughterhouses, live-pig transport hubs, and wildlife habitats, and continued development of assembly algorithms for hypervariable circovirus sequences—would improve the chance of detecting a future PCV6 early [24]. Additional CRESS DNA viruses, such as porcine circovirus-like viruses with ~3.9 kb genomes, have already been detected in swine [18], so the boundaries of this viral group are unlikely to be closed.

### 7.2. Implications for Swine Disease Control and Prevention

The etiology of porcine circovirus-associated disease continues to widen. Control measures have long centered on PCV2; the emergence of PCV3 broadened the etiological spectrum [82], and the discovery of PCV5 adds a further layer. Because these types are antigenically distant, existing PCV2 and PCV3 vaccines do not cross-protect against PCV5 [18,23]. Surveillance in South China found a herd-level PCV5 positivity rate of 24.3% (17/70 farms across Guangdong, Guangxi, Hunan, Yunnan, and Hainan provinces), a clinical sample positivity rate of 54.4% (294/540), and a seroprevalence of 66.8%, rising to 71.4% in breeding sows [18]. Against mostly inapparent clinical signs, seroprevalence at that level suggests that PCV5 is already circulating subclinically in breeding herds, and co-infection with pathogens such as PRRSV or PCV2 could compound the immunosuppression further [18]. Then came a second, independent survey in 2026. Researchers at Henan Agricultural University tested 100 clinical samples collected from 27 pig farms in seven provinces, obtaining a sample positivity rate of 22% (22/100), with the positives concentrated in Henan (21/82) and Fujian (1/2) [19]. Fecal samples predominated among the positives, an observation that marks the fecal–oral route as a key transmission pathway [19]. Co-infection proved to be the rule rather than the exception. Of the 22 positive samples, 18 carried additional swine pathogens, PCV2, PCV3, and PEDV included, and only 4 were PCV5 monoinfections. Evidence of that composition warrants caution in any claim about primary pathogenicity [19]. Taken together, the two surveys, one from South China and one from Central China and complementary in geography and sample type, outline the epidemiology of PCV5 in Chinese pig herds as one of high prevalence, frequent co-infection, and pathogenicity still unproven.

An indirect ELISA for PCV5 based on VLPs has already been developed. Its sensitivity and specificity are good, and because the assay avoids the cross-reactivity inherent in whole-virus antigens [18], veterinarians gain a standardized instrument for profiling herd immunity and detecting infection early. PCV5 viral load, moreover, correlates positively with clinical disease severity, although the relationship remains correlational rather than causal [18]. A practical path follows from these properties. Farms could move beyond qualitative detection into dynamic viral-load monitoring, identify and cull the heaviest virus shedders, and shift PCV5 control away from passive diagnosis and toward active surveillance with biosecurity-based elimination.

### 7.3. Concluding Remarks

Fifty years passed between the recognition of PCV1 as a cell culture contaminant in 1974 and the characterization of PCV5 in 2025. Throughout that span the group has continuously evolved via two major mechanisms, point mutations fine-tuning sequences and recombination remodeling genomes, and it proved ecologically resilient the whole way, absorbing wave after wave of emergence. With PCV3 and PCV4 in the mid-2010s, and then PCV5 in 2025, the picture changed in a further respect. Previously regarded as a single diversifying lineage, this group is now recognized as a polyphyletic assemblage, several of whose members entered the swine host independently and from unrelated backgrounds. The phylogenetic link between PCV5 and FSfaCV would, if the testable hypothesis sketched in Section 5.3 is borne out, extend this picture from terrestrial mammals toward marine or coastal ecosystems—pending identification of the true reservoir and transmission route—and in any case illustrates how large and dynamic the reservoir of CRESS DNA viruses is [18,20,26]. Once the ICTV rules on the proposed reclassification of PCV5, the nomenclature of this virus should be updated accordingly. Beyond taxonomy, the inverted transcriptional polarity of the two major PCV5 ORFs—Cap on the sense strand and Rep on the antisense strand—distinguishes PCV5 from all classical PCVs at the level of genome organization [19], and argues against treating the PCV assemblage as a single evolutionary unit even on structural grounds.

Against a pathogen with this degree of evolutionary plasticity, swine-centric surveillance frameworks are no longer sufficient. We advocate, under the One Health paradigm, surveillance of the CRESS DNA virome across the human–livestock–wildlife–environment interface, integrating environmental science, marine biology, molecular evolutionary biology, and wildlife ecology. Building early-warning capacity before the next host jump occurs would shift swine disease prevention from reactive mitigation to active defense.

## Figures and Tables

**Table 1:** Comparison of basic characteristics among PCV1–PCV5.

Characteristics	PCV1	PCV2	PCV3	PCV4	PCV5
First reported	1974/1982	Late 1990s	2016	2019	2025
Genome size	~1.76 kb	~1.77 kb	~2.0 kb	~1.7 kb	2.9 kb
Rep (aa)	312	314	297	296	351 (1056 nt)
Cap (aa)	233	233	214	228	~394 (1182 nt)
Stem-loop structure	Conserved (nonanucleotide motif within loop)	Conserved (nonanucleotide motif within loop)	Conserved (nonanucleotide motif within loop)	Conserved (nonanucleotide motif within loop)	Stem 9 nt/Loop 17 nt, extended
Phylogenetic position	Within *Circoviridae*	Within *Circoviridae*	Within *Circoviridae*	Within *Circoviridae*	Outside *Circoviridae*, closely related to FSfaCV; genus Macochavirus (assignment of PCV5 proposed) [20]
Associated diseases	Non-pathogenic	PCVAD/PMWS	PDNS, reproductive failure	PDNS-like diseases, etc.	Detected in pigs with respiratory disease, diarrhea, and reproductive disorders (causality unproven)

**Table 2:** Comparison of key domains of Rep and Cap proteins among PCV1–PCV5.

Protein/Domain	PCV1–PCV4	PCV5
Rep length	296–314 aa	351 aa
Rep three-domain architecture (Endonuclease/Oligomerization/SF3 Helicase)	Conserved	Conserved (structural prediction and biochemical validation)
RCR and Walker motifs	Conserved	Conserved (exhibits ATPase activity)
Cap length	214–233 aa	~394 aa
N-terminal arginine-rich NLS	Present	Present (predicted)
Dominant neutralizing epitopes	Loop regions (CD, HI, etc.); PCV2 harbors a decoy epitope	To be identified

**Table 3:** Chronology of the major PCV2 genotypes, with their principal characteristics.

Genotype	Epidemic Period	Key Characteristics
PCV2a	1990s–2003	Initial predominant genotype; major origin of current commercial vaccine strains
PCV2b	2003–2013	First global genotypic replacement, synchronized with the widespread PMWS pandemic
PCV2c	Retrospective detection	Retrospectively identified in archived Danish samples from the 1980s; currently found sporadically in *wild boars* in Brazil and *domestic* pigs in China
PCV2d	2013–present	Current global dominant genotype; Cap carries characteristic motifs such as SNPLSV (residues 86–91); current vaccines provide only limited cross-protection against it
PCV2g	Regional epidemic	Observed in regions such as Southeast Asia, mostly acting as a minor genotype
PCV2h	Regional epidemic (Vietnam, etc.)	Resulting from inter-genotypic recombination among PCV2a/2b/2d; highly associated with local Vietnamese vaccine strains

Note: Rare genotypes (PCV2e, PCV2f) are omitted from this timeline; they have been detected only sporadically at low frequency.

**Table 4:** Evidence assembled for cross-host transmission of the different PCV genotypes.

PCV Genotype	Primary Host	Reported Non-Swine Hosts	Level of Evidence
PCV1	Swine (cell culture contaminant)	Primates (including vaccine contamination incidents)	Nucleic acid detection
PCV2	*Sus scrofa domesticus*, *Sus scrofa*	Cattle, dogs, raccoon dogs, foxes, mice	Nucleic acid detection, virus isolation, and pathogenic evidence
PCV3	*Sus scrofa domesticus*, *Sus scrofa*	Cattle, dogs, deer, ticks	Extensive molecular epidemiological and serological evidence
PCV4	*Sus scrofa domesticus*, *Sus scrofa*	Cattle, dogs, raccoon dogs, foxes	Multi-species nucleic acid detection
PCV5	Swine (currently)	Unreported	Phylogenetic inference (closely related to FSfaCV)

## Data Availability

No new data were created or analyzed in this study. Data sharing is not applicable to this article.

## Abbreviations

The following abbreviations are used in this manuscript:

BRDC	bovine respiratory disease complex
Cap	capsid protein
CRESS	Circular Rep-encoding single-stranded DNA
FSfaCV	fur seal feces-associated circular DNA virus
HBV	hepatitis B virus
HPD	highest posterior density
HS	heparan sulfate
HSV-1	herpes simplex virus 1
ICTV	International Committee on Taxonomy of Viruses
NLS	nuclear localization signal
ORF	open reading frame
PCV	porcine circovirus
PCVAD	porcine circovirus-associated disease
PEDV	porcine epidemic diarrhea virus
PDNS	porcine dermatitis and nephropathy syndrome
PMWS	postweaning multisystemic wasting syndrome
PMV	porcine Macochavirus
PRRSV	porcine reproductive and respiratory syndrome virus
Rep	replication-associated protein
SF3	superfamily 3
SPF	specific pathogen free
VLP	virus-like particle
